# Neoadjuvant Therapy for Resectable and Borderline Resectable Pancreatic Cancer: A Meta-Analysis of Randomized Controlled Trials

**DOI:** 10.3390/jcm9041129

**Published:** 2020-04-15

**Authors:** Jordan M. Cloyd, Victor Heh, Timothy M. Pawlik, Aslam Ejaz, Mary Dillhoff, Allan Tsung, Terence Williams, Laith Abushahin, John F. P. Bridges, Heena Santry

**Affiliations:** 1 Division of Surgical Oncology, The Ohio State Wexner Medical Center, Columbus, OH 43210, USA; 2 Radiation Oncology, The Ohio State Wexner Medical Center, Columbus, OH 43210, USA; 3 Internal Medicine, The Ohio State Wexner Medical Center, Columbus, OH 43210, USA; 4 Biomedical Informatics, The Ohio State Wexner Medical Center, Columbus, OH 43210, USA

**Keywords:** preoperative therapy, pancreatic cancer, pancreas cancer, chemotherapy, radiation therapy, folfirinox, pancreatectomy

## Abstract

The efficacy of neoadjuvant therapy (NT) versus surgery first (SF) for pancreatic ductal adenocarcinoma (PDAC) remains controversial. A random-effects meta-analysis of only prospective randomized controlled trials (RCTs) comparing NT versus SF for potentially resectable (PR) or borderline resectable (BR) PDAC was performed. Among six RCTs including 850 patients, 411 (48.3%) received NT and 439 (51.6%) SF. In all included trials, NT was gemcitabine-based: four using chemoradiation and two chemotherapy alone. Based on an intention-to-treat analysis, NT resulted in improved overall survival (OS) compared to SF (HR 0.73, 95% CI 0.61–0.86). This effect was independent of anatomic classification (PR: hazard ratio (HR) 0.73, 95% CI 0.59–0.91; BR: HR 0.51 95% CI 0.28–0.93) or NT type (chemoradiation: HR 0.77, 95% CI 0.61–0.98; chemotherapy alone: HR 0.68, 95% CI 0.54–0.87). Overall resection rate was similar (risk ratio (RR) 0.93, 95% CI 0.82–1.04, I^2^ = 39.0%) but NT increased the likelihood of a margin-negative (R0) resection (RR 1.51, 95% CI 1.18–1.93, I^2^ = 0%) and having negative lymph nodes (RR 2.07, 95% CI 1.47–2.91, I^2^ = 12.3%). In this meta-analysis of prospective RCTs, NT significantly improved OS in an intention-to-treat fashion, compared with SF for localized PDAC. Randomized controlled trials using contemporary multi-agent chemotherapy will be needed to confirm these findings and to define the optimal NT regimen.

## 1. Introduction

Despite recent improvements, pancreatic ductal adenocarcinoma (PDAC) remains a deadly cancer with an overall survival (OS) rate at 5 years of only 10% [1]. Even patients with localized cancers who undergo resection are still likely to experience recurrence [2]. Adjuvant chemotherapy following surgery improves OS rates [3,4,5]. However, a significant proportion of patients are unable to initiate adjuvant chemotherapy following pancreatectomy, frequently because of postoperative complications or rapid disease recurrence [6]; many more are unable to finish the planned course of adjuvant therapy [7]. Indeed, a strategy of surgery first (SF) followed by planned adjuvant therapy has not resulted in significant improvements in OS during the past several decades, even at high-volume, experienced institutions [8,9].

In contrast, the administration of chemotherapy and/or radiation therapy prior to surgery guarantees the delivery of systemic therapies. In retrospective analyses, neoadjuvant therapy (NT) has been associated with improved rates of margin-negative resection and a decreased incidence of lymph node metastases [10,11]. NT also offers several other theoretical benefits, including early treatment of presumed micro-metastatic disease, enhanced selection of patients with appropriate tumor biology for surgery, and the ability to histologically measure the response to therapy [12,13]. Evidence of improved survival has also been suggested based on data from cancer databases [10], meta-analyses of retrospective studies [14], and Markov decision models [15]. In turn, NT has become the preferred approach for borderline resectable (BR) PDAC, while guidelines support the use of either SF or NT for potentially resectable (PR) PDAC [16,17,18].

Despite the theoretical and empirical advantages of NT, its use in the United States has remained relatively low [19,20], potentially driven by the lack of level I evidence for its efficacy. Until recently, only two small randomized controlled trials (RCTs) had been performed comparing SF to NT, and both were terminated early due to poor accrual [21,22]. Previous systematic reviews and meta-analyses that have purported a survival benefit with NT included non-randomized prospective and retrospective studies, which are limited due to their inherent selection biases [14,23,24,25,26]. As several larger RCTs have recently been completed, albeit with older neoadjuvant regimens, the purpose of the current study was to perform a meta-analysis limited to only RCTs evaluating SF vs. NT for PDAC.

## 2. Materials and Methods

### 2.1. Literature Search Strategy

The meta-analysis was performed according to Preferred Reporting Items for Systematic Reviews and Meta-Analyses (PRISMA) guidelines [27,28]. A systematic review of the Pubmed, Cinahl, Medline, Embase, and CENTRAL databases was performed using the following Medical Subject Heading (MeSH) search terms: pancreatic cancer, neoadjuvant therapy, and randomized controlled trial. Multiple combinations of search terms were used. The final search was completed on 31 January 2020 and updated on 25 March 2020; the search strategy is reported in Table S1. Screening was performed using Covidence (Melbourne, Australia).

Studies including patients with PR or BR PDAC who were randomized to either SF or neoadjuvant chemotherapy and/or radiation therapy, and reported OS as a primary or secondary outcome, were included. Studies involving locally advanced (LA) PDAC were excluded. Retrospective and non-randomized prospective studies, and those without English-language abstracts, were excluded while published conference abstracts were not.

All titles, abstracts, and full texts were reviewed for inclusion according to the above criteria. Data extraction from the included studies was performed by two authors, and disagreements were resolved with discussion until consensus was achieved. Data on number of patients, resection rate, margin-negative (R0) resection rate, lymph node positivity rate, grade ≥3 complications, OS duration or hazard ratio, and type of adjuvant therapy were recorded for both treatment arms; type of NT, grade ≥3 serious adverse events (SAE) during NT, and completion rate were recorded for patients undergoing NT. All studies were assessed for risk of bias according to the Cochrane ROB-2 tool from the Cochrane Collaboration [29]. The quality of evidence for each outcome was estimated according to the Grading of Recommendations Assessment, Development and Evaluation (GRADE) process [30].

### 2.2. Statistical Analysis

The primary outcome was OS on an intention-to-treat basis, while secondary outcomes were overall resection rate (intention-to-treat) and R0 resection rate (among patients who underwent resection). Hazard ratios (HRs) and risk ratios (RRs) were used as effect size for each respective outcome. For the primary outcome, HRs were extracted from articles in which these data were provided. In instances where HRs were not reported, HRs and 95% confidence intervals (95% CIs) were estimated using indirect methods, based on observed events, total events, log-rank or Cox proportional model *p*-values and published Kaplan–Meier curves [31]. For the secondary outcome, RR and 95% CI were estimated based on event rates reported in 2 × 2 table formats.

RRs and HRs (95% CI) were log-transformed, and standard errors were computed based on log-transformed confidence interval bounds. Pooled logRR and logHR were then calculated using random-effects models via the restricted maximum likelihood estimator (REML). REML is approximately unbiased and efficient [32]. Median OS was estimated for each group via the following methodology [33]: log-transformed survival times (logMST) from each study were pooled in each treatment group, using weights derived from variances resulting from both sample size in each group and study precision of HRs. The standard error of the combined logMST was then calculated as the square root of the sum of the inverse variance weights.

Sensitivity analysis based on the random-effects model was done to examine effects of setting (PR vs. BR) and NT type (chemotherapy vs. chemoradiation (CRT)) on the primary outcomes by analyzing the relevant trials. HR < 1 for OS or RR > 1 for the secondary outcomes where the 95% CI does not contain 1 would favor NT. The amount of true heterogeneity in logRR or logHR was assessed using the *I^2^* statistic. For ease of interpretation, estimated average logRR and logHR and 95% CIs were transformed back to RR and HR scales through exponentiation and results displayed using forest plots. All meta-analyses were done using R package (metafor).

## 3. Results

**Figure 1:**
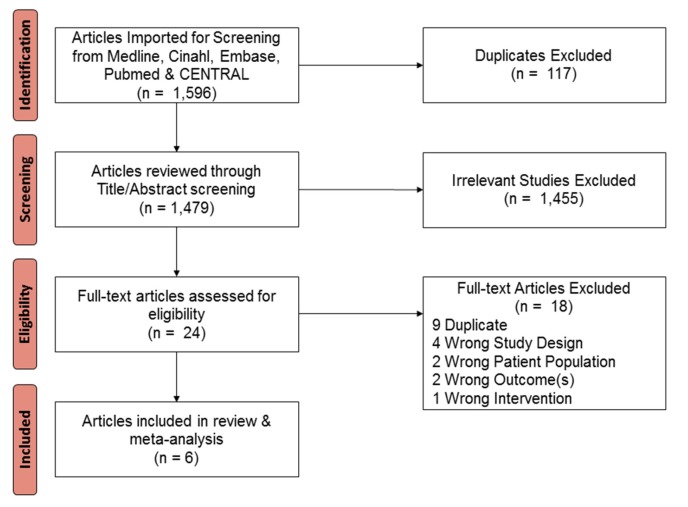
Flow diagram of study selection using Preferred Reporting Items for Systematic Reviews and Meta-Analyses (PRISMA) guidelines.

Among 1586 articles identified, 24 were selected for full-text review, and 6 RCTs comparing SF to NT for PDAC were included (Figure 1) [21,22,34,35,36,37]. The characteristics of the included trials are reported in Table 1. A total of 850 patients were included: 411 (48.3%) received NT and 439 (51.6%) SF. Of the six trials, four included patients with PR disease, one with BR tumors, and one included PR and BR tumors. Similarly, four trials used neoadjuvant CRT while two used systemic chemotherapy alone. All NT was gemcitabine-based; none utilized mFOLFIRINOX or gemcitabine/nab-paclitaxel. All included trials also recommended adjuvant therapy; five regimens were gemcitabine-based and one used S-1. Results of the methodological assessment are reported in Figure S1; none was assessed as having a high risk of bias.

**Figure 2:**
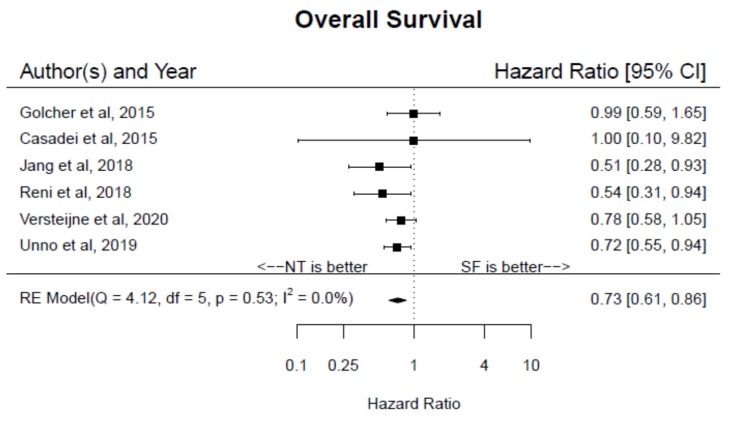
Forest plot of pooled hazard ratio for overall survival among patients with pancreatic cancer randomized to neoadjuvant therapy versus surgery first.

The primary and secondary outcomes from the six included trials are summarized in Table 2. Based on an intention-to-treat analysis, the pooled HR for OS of NT compared to SF was 0.73 (95% CI 0.61–0.86, I^2^ = 0%) (Figure 2). Similarly, the pooled median OS across all studies was greater among patients receiving NT than SF (25.4 months (95% CI 22.4–28.7) vs. 19.4 (95% CI 17.2–21.8), *p* < 0.001). In pre-determined subset analyses, the pooled HR remained significantly in favor of NT independent of anatomic classification (PR: HR 0.73, 95% CI 0.59–0.91; BR: HR 0.51 95% CI 0.28–0.93) or neoadjuvant treatment type (CRT: HR 0.77, 95% CI 0.61–0.98; chemotherapy alone: HR 0.68, 95% CI 0.54–0.87).

**Figure 3:**
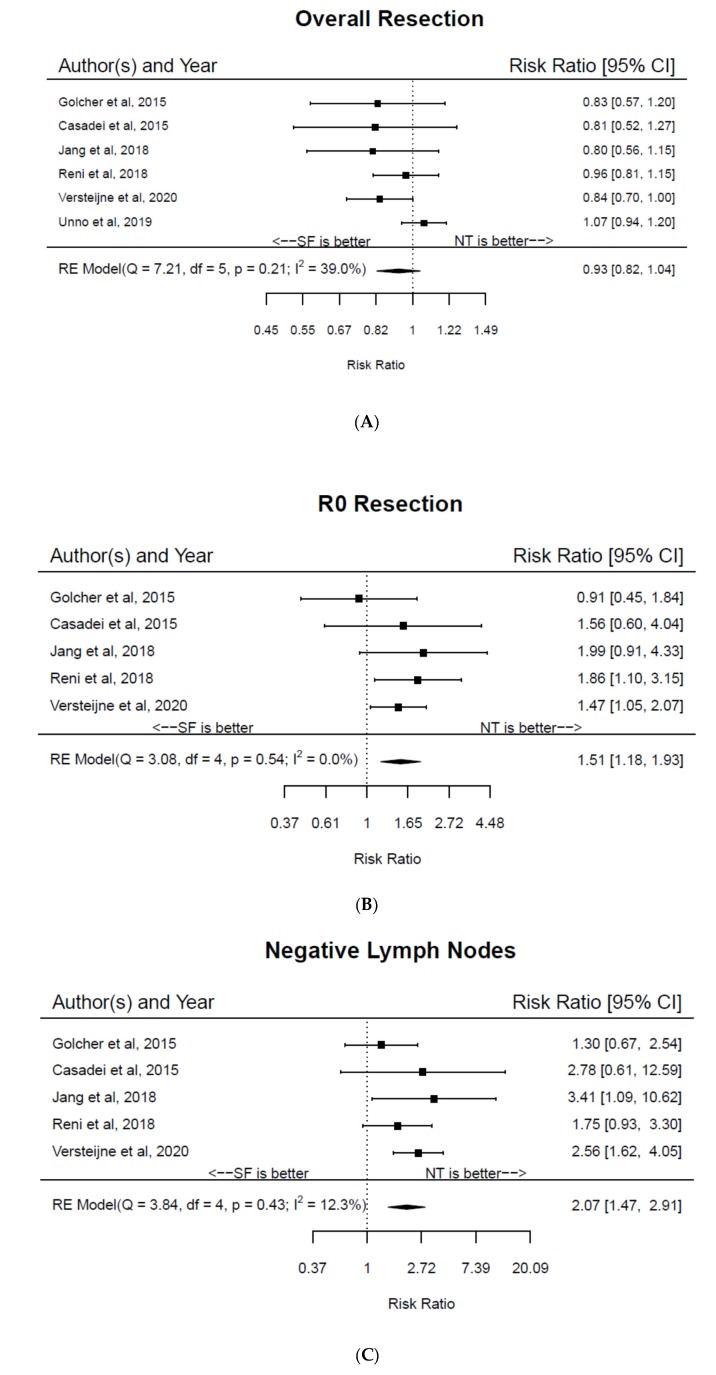
Forest plots of pooled relative risk ratios for (**A**) overall resection rates, (**B**) R0 resection rates, and (**C**) lymph node negative rates among patients with pancreatic cancer randomized to neoadjuvant therapy versus surgery first.

Five trials reported adverse event rates during NT with SAE rates ranging from 11.1% to 72.0%. On an intention-to-treat basis, there was no significant difference in overall resection rates among the two groups (RR 0.93, 95% CI 0.82–1.04, I^2^ = 39.0%) (Figure 3A). On the other hand, among patients who underwent resection, NT increased the likelihood of an R0 resection (RR 1.51, 95% CI 1.18–1.93, I^2^ = 0%) (Figure 3B) and increased the pathologic lymph node negative rate (RR 2.07, 95% CI 1.47–2.91, I^2^ = 12.3%) (Figure 3C). Three studies reported grade ≥3 morbidity rates following surgery with rates ranging from 11.1% to 31.56% among patients who received NT and from 16.7% to 65.2% among those who underwent SF. The overall quality of evidence according to GRADE was felt to be moderate (Table S2). 

## 4. Discussion

In this meta-analysis of only prospective RCTs, the OS of patients with resectable or borderline resectable PDAC who received NT was nearly 30% greater than that of patients who underwent immediate surgery using an intention-to-treat design. These findings remained significant among both patients with PR and BR disease and were independent of the type of NT. The current study also confirmed improvements in R0 resection rate and lymph node positivity with the use of NT and no difference in overall resection rate. Taken together, these results represent the strongest evidence to date for NT in the management of localized PDAC.

Immediate surgical resection followed by adjuvant chemotherapy has long been the standard treatment for PDAC, and recent trials suggest improving OS for those patients who are able to undergo resection and receive adjuvant therapy [4,5,38]. However, pancreatectomy is a complex, potentially morbid operation, and a significant proportion of patients are unable to receive adjuvant therapy either due to postoperative complications or poor performance status [6,7]. In addition, many patients develop rapid disease progression in the immediate postoperative period, suggesting that surgery was not only not beneficial for these patients, but potentially detrimental in delaying needed systemic therapy [39]. Administering non-surgical therapy prior to pancreatectomy would not only ensure its delivery and improve patient selection for surgery, but has also been theorized to improve complete microscopic tumor resection and locoregional control [12].

Early RCTs evaluating the role of NT for PDAC were unsuccessful. Both Golcher et al. and Casadei et al. intended to evaluate the use of preoperative CRT for patients with PR PDAC, but both trials were closed early due to poor patient accrual with no significant differences observed in OS [21,22]. Jang et al. performed a multicenter Korean RCT comparing neoadjuvant CRT to surgery first for BR PDAC. Based on the findings of improved OS and R0 resection rate, the trial was terminated early [37]. Similarly, Reni et al. performed a three-arm RCT comparing immediate surgery and two different adjuvant chemotherapy regimens to neoadjuvant chemotherapy followed by surgery and adjuvant therapy for patients with resectable PDAC. Based on the observed improved OS, the planned phase III component was not continued [34]. These first four RCTs highlight not only the challenges in designing, conducting, accruing, and completing trials in PDAC, but also the strong desire for novel treatment options with stronger evidence for this aggressive cancer. More recently, the PREOPANC trial randomized patients with PR and BR PDAC to either neoadjuvant gemcitabine-based CRT or SF; while the primary outcome OS was non-significant (*p* < 0.1), several of the secondary endpoints and predefined subgroup analyses were encouraging [36]. Finally, Prep-02/JSAP-05 was a large multicenter Japanese RCT comparing neoadjuvant gemcitabine and S-1 with SF in patients with resectable PDAC. Although presented in conference form only, NT led to significantly improved OS compared to SF (36.7 vs. 26.6 months; *p*=0.02) [35].

While recent RCTs and the current meta-analysis provide strong evidence to support the use of NT, many questions remain, particularly regarding the optimal components of therapy. Early trials primarily focused on preoperative CRT [40,41,42]; however, as the chemotherapy options for PDAC have improved, increasingly chemotherapy alone is being used prior to surgery, especially for patients with PR disease [43]. Nevertheless, the best chemotherapy regimen has not been established, and it is unlikely that the chemotherapy regimens used in the PACT-15 (Cisplatin, Epirubicin, Gemcitabine, Capecitabine) or Prep-02/JSAP-05 (gemcitabine, S-1) will be widely adopted [34,35]. Given that most patients currently receive either gemcitabine/nab-paclitaxel or mFOLFIRINOX, the ongoing NEONAX [44], NEPAFOX [45], NORPACT-1 [46], PANACHE01-PRODIGE48 [47], PREOPANC-2 [48], ESPAC-5 [49], and SWOG S1505 [50] trials as well as the upcoming Alliance A021806 trial, which incorporate one of these regimens, should improve our understanding of the preferred preoperative regimen.

In the era of more effective systemic chemotherapy, the role of CRT in the management of PDAC remains poorly understood. The four included RCTs and the pooled analysis in the current study support the use of neoadjuvant CRT compared to SF for patients with PR and BR PDAC. Compared to chemotherapy alone, retrospective studies have suggested that CRT is associated with improved margin-negative resection rates and locoregional control [51,52]. However, few prospective trials have compared chemotherapy alone to CRT, and thus the ongoing PREOPANC-2 and ESPAC-5 will be informative [48,49]. An important question remains regarding the role of neoadjuvant radiation therapy following induction systemic chemotherapy, particularly for patients with BR and LA disease [53]. The ongoing Alliance A021501 trial, comparing mFOLFORINOX with or without stereotactic body radiation therapy, may provide new insights [54].

As the prior literature on the efficacy of NT has largely been based on retrospective studies, many investigators have posited that any benefits observed when comparing NT and SF must occur due to the patient selection afforded by NT. However, the results of the current intention-to-treat meta-analysis (which include those patients who were unable to undergo curative-intent surgery) suggest that these findings may represent true treatment benefits. Despite the importance of these findings, several limitations should be acknowledged. First, the study included both PR and BR cancers, which have different anatomic characteristics and were not uniformly defined in the included trials. Second, the NT treatment types were heterogeneous and none included contemporary Western chemotherapy regimens more commonly used today. Third, one included trial has only been published in conference form, and our methodology was unable to use individual patient-level data [55]. Fourth, none of the randomized controlled trials included in this meta-analysis were blinded to the receipt of NT, potentially leading to a bias in the decision to proceed with resection in the neoadjuvant cohorts; since LN negative and R0 resection rates were only calculated among those who undergo resection, this could potentially confound these secondary outcomes. Finally, while minimal statistical heterogeneity was observed in the current meta-analysis, this could be an underestimate given the small number of included studies [56]; random-effects modeling was used regardless to account for possible heterogeneity.

In conclusion, in the first meta-analysis of NT versus SF for localized PDAC using only prospective RCTs, NT significantly improved OS in an intention-to-treat fashion. These results suggest that NT should be the preferred treatment strategy for patients with BR PDAC and can be considered for patients with PR PDAC. However, the included studies were largely based on outdated neoadjuvant regimens. Randomized controlled trials using contemporary multi-agent chemotherapy will be needed to confirm these findings and to define the optimal NT regimen.

## Figures and Tables

**Table 1:** Study characteristics for randomized controlled trials included in the meta-analysis.

Author	Institution	Origin	Setting	PR/BR Definition	Sample Size	Neoadjuvant Therapy	Regimen	Adjuvant Therapy
Golcher	Multi-	Germany	PR	≤180 “peripancreatic vessels”	66	CRT	Gemcitabine/Cisplatin; 56Gy	Gemcitabine
Casadei	Single-	Italy	PR	<180 SMV/PV; No contact to CA/HA/SMA	38	CRT	Gemcitabine; 54Gy	Gemcitabine
Jang	Multi-	Korea	BR	2012 NCCN criteria	50	CRT	Gemcitabine; 54Gy	CRT, Gemcitabine
Reni	Multi-	Italy	PR	No invasion of SMA/SMV/PV/CA/HA	88	Chemo	Cisplatin, Epirubicin, Gemcitabine, Capecitabine	Cisplatin, Epirubicin, Gemcitabine, Capecitabine or Gemcitabine
Versteijne	Multi-	Netherlands	PR/BR	PR: <90 SMV/PV; no CA/HA/SMA contact BR: <90 CA/HA/SMA; 90–270 PV/SMV without occlusion	246	CRT	Gemcitabine; 36Gy	Gemcitabine
Unno	Multi-	Japan	PR	No CA/HA/SMA abutment	362	Chemo	Gemcitabine, S-1	S1

PR, potentially resectable; BR, borderline resectable; CRT, chemoradiation; SMV, superior mesenteric vein; PV, portal vein; CA, celiac axis; HA, hepatic artery; SMA, superior mesenteric artery; NCCN, National Comprehensive Cancer Network.

**Table 2:** Outcomes of randomized controlled trials included in the meta-analysis.

Author	SAE Rate (%)	Resected Rate (%)		R0 Resection Rate (%)		pN0 Rate (%)		Grade ≥3 Postoperative Morbidity (%)		Overall Survival (mo)	
	NT	NT	SF	NT	SF	NT	SF	NT	SF	NT	SF
Golcher	45.5	57.6	69.7	52.6	47.8	68.4	43.5	31.56	65.2	17.4	14.4
Casadei	22.2	61.1	75.0	63.6	33.3	45.5	13.3	N/A	N/A	22.4	19.5
Jang	11.1	63.0	78.3	82.4	33.3	70.6	16.7	23.5	16.7	21.0	12.0
Reni	34.4	84.4	87.5	63.0	32.7	48.1	26.5	11.1	20.4	38.2	20.4–26.4
Versteijne	N/A	60.5	72.4	70.8	40.2	66.7	21.7	68.1 *	50.0 *	16.0	14.3
Unno	72.0	76.9	72.2	N/A	N/A	N/A	N/A	N/A	N/A	36.7	26.6

SAE, serious adverse events; NT, neoadjuvant therapy; SF, surgery first; N/A, not applicable; * Any complication.

## Supplementary Materials

The following are available online at https://www.mdpi.com/2077-0383/9/4/1129/s1. Figure S1: Risk of bias assessment according to Cochrane Collaboration ROB-2 tool, Table S1: Search terms used in systematic literature review, Table S2: Results of Cochrane Collaboration risk of bias assessment.
