# Supplementary material for: Neoadjuvant Therapy for Resectable and Borderline Resectable Pancreatic Cancer: A Meta-Analysis of Randomized Controlled Trials
Source: J Clin Med. 2020 Apr 15;9(4):1129. doi: 10.3390/jcm9041129 (PMC7231310; doi:10.3390/jcm9041129)
Supplement: Supplementary file 1 [file jcm-09-01129-s001.pdf]

Table S1. Search terms used in systematic literature review.

Database	Search Strategy
Pubmed	("randomized control trial" OR "random control trial" OR (("randomized" OR "randomised") AND "open-label" AND ("trial" OR "study")) OR "RCT" OR (("random" OR "randomized") AND "control" AND ("trial" OR "study")) OR "random control study" OR "randomized control study") AND (("neoadjuvant therapy"[MeSH Terms] OR ("neoadjuvant"[All Fields] AND "therapy"[All Fields]) OR "neoadjuvant therapy"[All Fields] OR "neoadjuvant chemoradiation" OR "neoadjuvant"[All Fields] OR "neoadjuvant treatment") OR "preoperative therapy"[All Fields] OR "pre-operative therapy"[All Fields] OR ("preoperative" AND "chemotherapy") OR downstaging[All Fields] OR "conversion therapy"[All Fields] OR ("adjuvants, pharmaceutic"[Pharmacological Action] OR "adjuvants, immunologic"[Pharmacological Action] OR "adjuvants, pharmaceutic"[MeSH Terms] OR ("adjuvants"[All Fields] AND "pharmaceutic"[All Fields]) OR "pharmaceutic adjuvants"[All Fields] OR "adjuvant"[All Fields] OR "adjuvant treatment"[All Fields] OR "adjuvants, immunologic"[MeSH Terms] OR ("adjuvants"[All Fields] AND "immunologic"[All Fields]) OR "immunologic adjuvants"[All Fields]) OR perioperative[All Fields] OR ("adjuvants, pharmaceutic"[Pharmacological Action] OR "adjuvants, immunologic"[Pharmacological Action] OR "adjuvants, pharmaceutic"[MeSH Terms] OR ("adjuvants"[All Fields] AND "pharmaceutic"[All Fields]) OR "pharmaceutic adjuvants"[All Fields] OR "adjuvant"[All Fields] OR "adjuvants, immunologic"[MeSH Terms] OR ("adjuvants"[All Fields] AND "immunologic"[All Fields]) OR "immunologic adjuvants"[All Fields]) OR presurgery[All Fields]) AND ("pancreatic cancer"[All Fields] OR (("pancreas"[MeSH Terms] OR "pancreas"[All Fields] OR "pancreatic"[All Fields]) AND ductal[All Fields] AND ("adenoids"[MeSH Terms] OR "adenoids"[All Fields] OR "adenoid"[All Fields]) AND ("carcinoma"[MeSH Terms] OR "carcinoma"[All Fields])) OR "pancreatic tumor"[All Fields] OR (("neoplasms"[MeSH Terms] OR "neoplasms"[All Fields] OR "cancer"[All Fields]) AND ("pancreas"[MeSH Terms] OR "pancreas"[All Fields])) OR "pancreatic neoplasms"[All Fields])
Cinahl	"neoadjuvant therapy" OR "neoadjuvant chemotherapy" OR "neoadjuvant chemoradiation" OR "neoadjuvant" OR ("neoadjuvant" AND "treatment") OR ("neoadjuvant" AND "therapy") OR "preoperative therapy" OR "pre-operative therapy" OR "preoperative treatment" OR ("preoperative" AND "chemotherapy") OR "presurgery" OR "preoperative" OR "preoperative period" OR "downstaging" OR "conversion therapy" OR "adjuvant therapy" OR "adjuvant" OR ("adjuvant" AND "treatment") OR "cancer immunotherapy" AND ("random control trial" or "rtc" or "randomized control trial" or "randomized control trial" or "randomized control study" OR (("randomized" OR "randomised") AND "open-label" AND "trial") AND ("pancreatic cancer" OR "pancreatic carcinoma" OR "carcinoma of pancreas" OR "pancreatic tumor" OR "pancreatic adenocarcinoma" OR (("pancreas" OR "pancreatic) AND "cancer") OR "pancreatic ductal adenocarcinoma" OR "pancreatic neoplasms")
Embase	('neoadjuvant therapy' OR 'neoadjuvant chemotherapy' OR 'neoadjuvant radiotherapy' OR 'neoadjuvant chemoradiotherapy' OR (neoadjuvant AND therapy) OR (neoadjuvant AND treatment) OR 'preoperative treatment' OR 'preoperative period' OR (preoperative AND chemotherapy) OR 'preoperative chemoradiotherapy' OR presurgery OR downstaging OR 'conversion therapy' OR 'adjuvant therapy' OR neoadjuvant OR adjuvant OR 'perioperative period' OR (perioperative AND treatment) OR (perioperative AND therapy) OR 'cancer immunotherapy') AND ('pancreas cancer' OR 'pancreas adenocarcinoma' OR 'pancreas tumor' OR (pancreas AND tumor) OR (pancreas AND cancer) OR 'pancreatic ductal

	adenocarcinoma' OR 'pancreas neoplasms' OR 'pancreatic cancer') AND ('randomized controlled trial' OR 'random control trial' OR 'randomized control study' OR ((randomized OR randomised) AND (trial OR 'clinical trial')))
CENTRAL	("neoadjuvant therapy" OR "neoadjuvant chemotherapy" OR "neoadjuvant chemoradiation" OR "neoadjuvant" OR ("neoadjuvant" AND "treatment") OR ("neoadjuvant" AND "therapy") OR "preoperative therapy" OR "pre-operative therapy" OR "preoperative treatment" OR ("preoperative" AND "chemotherapy") OR "presurgery" OR "preoperative" OR "preoperative period" OR "downstaging" OR "conversion therapy" OR "adjuvant therapy" OR "adjuvant" OR ("adjuvant" AND "treatment") OR "cancer immunotherapy") AND ("random control trial" or "rtc" or "randomized control trial" or "randomized control trial" or "randomized control study" OR (("randomized" OR "randomised") AND "open-label" AND "trial")) AND ("pancreatic cancer" OR "pancreatic carcinoma" OR "carcinoma of pancreas" OR "pancreatic tumor" OR "pancreatic adenocarcinoma" OR (("pancreas" OR "pancreatic") AND "cancer") OR "pancreatic ductal adenocarcinoma" OR "pancreatic neoplasms")

Table S2. Quality of evidence assessment according to GRADE criteria.

Outcome	Quality of Evidence
Overall Survival	Moderate
Resection Rate	Low
R0 Resection	Moderate
pN0 Rate	High

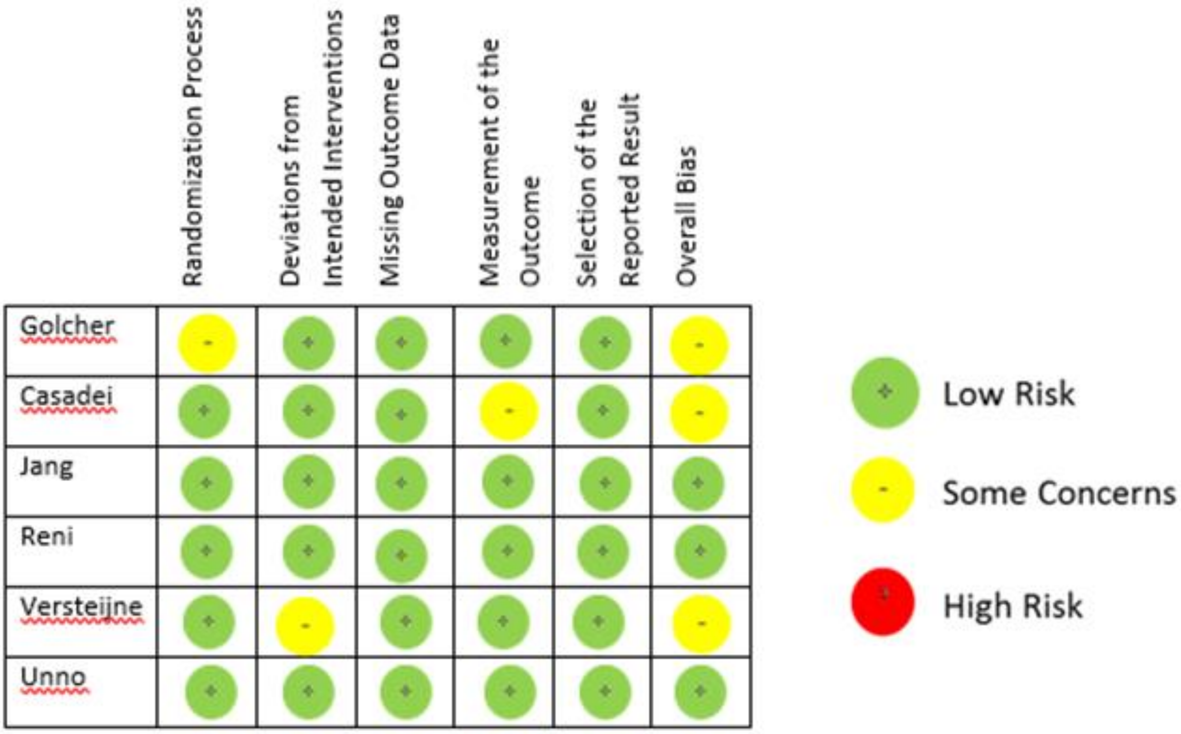

Figure S1. Risk of bias assessment according to Cochrane Collaboration ROB-2 tool.
